# Cancer metastasis as a non-healing wound

**DOI:** 10.1038/s41416-021-01309-w

**Published:** 2021-03-17

**Authors:** Matthew Deyell, Christopher S. Garris, Ashley M. Laughney

**Affiliations:** 1 Institute for Computational Biomedicine, Weill Cornell Medicine, New York, NY USA; 2 Department of Physiology and Biophysics, Weill Cornell Medicine, New York, NY USA; 3 Sandra and Edward Meyer Cancer Center, Weill Cornell Medicine, New York, NY USA; 4 Chimie Biologie et Innovation, ESPCI Paris, Université PSL, CNRS, Paris, France; 5 Rockefeller University, New York, NY USA

**Keywords:** Tumour heterogeneity, Metastasis, Cancer microenvironment, Cancer stem cells

## Abstract

Most cancer deaths are caused by metastasis: recurrence of disease by disseminated tumour cells at sites distant from the primary tumour. Large numbers of disseminated tumour cells are released from the primary tumour, even during the early stages of tumour growth. However, only a minority survive as potential seeds for future metastatic outgrowths. These cells must adapt to a relatively inhospitable microenvironment, evade immune surveillance and progress from the micro- to macro-metastatic stage to generate a secondary tumour. A pervasive driver of this transition is chronic inflammatory signalling emanating from tumour cells themselves. These signals can promote migration and engagement of stem and progenitor cell function, events that are also central to a wound healing response. In this review, we revisit the concept of cancer as a non-healing wound, first introduced by Virchow in the 19th century, with a new tumour cell-intrinsic perspective on inflammation and focus on metastasis. Cellular responses to inflammation in both wound healing and metastasis are tightly regulated by crosstalk with the surrounding microenvironment. Targeting or restoring canonical responses to inflammation could represent a novel strategy to prevent the lethal spread of cancer.

## Background

Since first proposed, the hallmarks of cancer have included metastasis—a critical and lethal trait acquired during the multistep evolution of cancer.^1^ This capability enables cancer to spread from the primary tumour to secondary sites within the body. Despite significant advances in our understanding of its molecular mechanisms, metastatic disease remains the leading cause of cancer-associated deaths.^2^ Many studies have detailed mechanisms leading to the formation of primary tumours; however, the invasion–metastasis cascade remains poorly understood due to its non-linear, multistep and dynamic nature. Broadly, the invasion–metastasis cascade spans four key phases.^3,4^ First, primary tumour cells must locally invade surrounding tissues. Secondly, tumour cells, including early disseminated cancer cells, break through the basal membrane and begin to circulate through blood or lymphatic vessels in a process called intravasation.^2,5–7^ Third, extravasation involves the exodus of often solitary cancer cells^2^ from the circulatory and lymphatic systems to a new microenvironment. To persist as potential seeds for future metastases, disseminated tumour cells (DTCs) must evade immune surveillance and resist anti-cancer therapies, which primarily target dividing cells.^3,8^ Only a minority of cells are able to withstand these stresses, retain metastasis-initiating potential, and ultimately progress from a micro-metastasis to the fourth and final phase of the invasion–metastasis cascade: secondary tumour formation (overt macro-metastases). This transition from a micro- to macro-metastasis is governed by cellular and physical microenvironmental crosstalk,^9–12^ tumour–immune interactions^13–16^ and dormancy.^17^

Notably, all processes within the invasion–metastasis cascade trigger inflammation and require breakdown and remodelling of the extracellular matrix (ECM)—events also central to a wound healing response. Similarities between cancer and wound healing have been apparent since Rudolph Virchow proposed his ‘irritation theory’ in 1858, in which he concluded that irritation and its subsequent inflammation were the essential factors that led to the formation of neoplastic tissues.^18,19^ Over a century later, these same similarities led Harold Dvorak to call tumours ‘wounds that do not heal’.^20^ However, the molecular mechanisms that underlie many reparative processes, including the ability to resolve inflammation, remain poorly understood in models of both injury and cancer. What has become clear is that wound healing is regulated by numerous positive and negative feedback mechanisms.^21^ Positive feedback mechanisms ensure that healing continues until the wound is fully resolved, as any partially restored tissue risks infection. This requires compensatory regeneration by progenitor cells to replace lost tissue mass. Likewise, negative feedback mechanisms turn off these processes once they are complete to prevent unchecked cellular growth. If left unresolved, persistent inflammation and cellular growth can lead to failed tissue repair and fibrosis.^22^

**Fig. 1:**
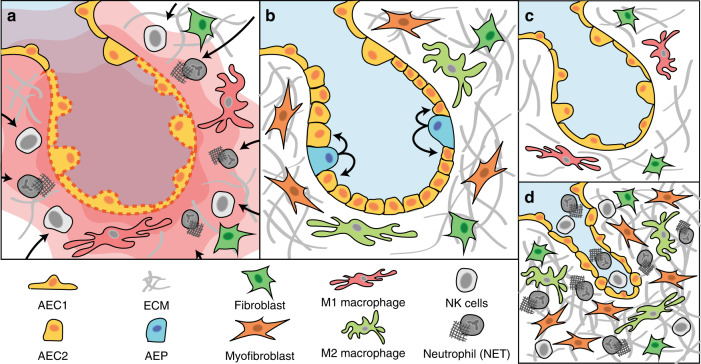
The wound-healing pathway. The wound healing process comprises three stages: inflammation, regeneration and remodelling. During inflammation (**a**), damage to epithelial cells (AEC1/AEC2) releases inflammatory signals (red waves). These signals cause immune cells such as neutrophils to migrate into the wound microenvironment to prevent infection and break down damaged tissue to initiate repair. Regeneration (**b**) of the wound begins when immune cells such as macrophages transition from a pro-inflammatory (M1) to an anti-inflammatory (M2) phenotype. This triggers the release of growth factors that engage the stem/progenitor functions of epithelial cells to replace cells that were damaged and lost. Concurrently, the surrounding extracellular matrix (ECM) is restored by macrophages, activated fibroblasts and myofibroblasts. **c** Remodelling completes the wound-healing pathway by removing excess ECM and cells. When remodelling fails (**d**), unchecked proliferation of immune cells, collagen and myofibroblasts causes them to persist in the wound microenvironment, leading to scarring and fibrosis.

**Fig. 2:**
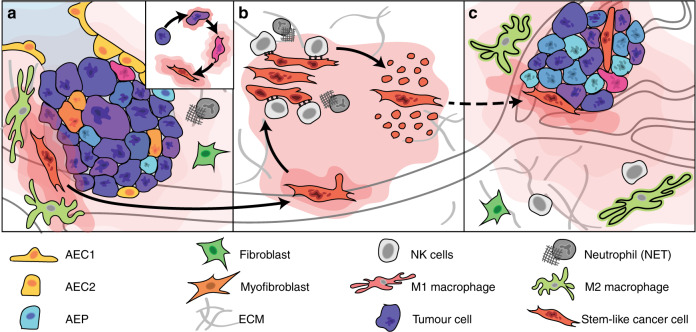
Cancer metastasis subverts mechanisms of the wound-healing pathway. Many mechanisms underlying inflammatory signalling, regeneration and unchecked proliferation can function in cancer progression, including during dissemination from the primary tumour, establishment of a micro-metastases, and finally overt macro-metastatic outgrowth. Chromosomal instability leads to the formation of rupture-prone micronuclei - a source of chronic inflammatory signalling in tumour cells themselves. At the invasive front of a primary tumour (**a**), chronic inflammatory signalling driven by sensing of cytosolic nucleic acids can trigger epithelial–mesenchymal transition (insert) and a more regenerative stem-like phenotype. Breakdown of the extracellular matrix (ECM) allows mesenchymal stem-like cells to escape the tumour microenvironment and seed micro-metastases (**b**). The innate immune system, especially natural killer (NK) cells, can restrict the expansion of latent, metastasis-initiating cells that have not yet adapted to evade the deleterious, immune-mediated consequences of chronic inflammatory signalling. Cells that enter an immune-evasive, quiescent state might eventually form overt macro-metastases under growth-permissive conditions (**c**), which can lead to re-engagement with pro-regenerative immune cells in the new tumour microenvironment.

Genomic instability triggers chronic inflammatory signalling from tumour cells themselves, which has recently emerged as a pervasive driver of metastases.^23^ Understanding how pathways that operate during wound healing are corrupted or co-opted in cancer to drive progression is therefore vital to our understanding and treatment of metastasis. In this review, we explore how the three overlapping wound healing stages—inflammation, regeneration and remodelling—are dysregulated and subverted to drive cancer metastasis, using the lung as a model system (Fig. 1). The lung displays facultative regeneration capabilities, whereby most of its epithelial cell types can be regenerated upon injury by progenitor sub-populations which engage with stem-like properties.^21,24^ Such an approach allows this relatively quiescent tissue to rapidly respond to frequent injury that may occur as a result of common triggers such as air pollutants or respiratory infections. This is in contrast to highly regenerative tissues such as the intestine, which have dedicated stem cell populations that homoeostatically regenerate,^25,26^ or non-regenerative tissues such as the heart or brain, which have limited healing capabilities.^27–29^ Lung metastases are a common cause of mortality across many primary tumour types, including melanoma, breast, lung and head and neck cancers.^30^ Importantly, the processes that drive metastasis to the lung, including genomic instability, have been demonstrated to be broadly applicable to other cancer types as well.^31,32^ Ultimately, the lung regeneration model allows us to decipher how the general feedback controls that operate in wound healing are dysregulated in cancer to drive the development of metastasis (Fig. 2). We begin our review by first exploring the role of inflammation in both normal wound healing and metastasis. We then proceed to the regeneration stage of the wound healing cascade and contrast it to the engagement of stem-like functions in metastasis. Finally, we discuss how resolution of the wound healing process through the remodelling stage is never reached, and the implications this has for both chronic wounds and metastatic cancers.

## Inflammation

Prior to the widespread use of antiseptics, infection of wounds was a common cause of morbidity, underscoring the critical interconnection between wound healing and immune responses. Control of infection is prioritised as one of the first responses to injury. This involves the recruitment of cell types that are responsible for clearing damaged tissues and infectious elements by inflammatory pathways.^21,33^ This response is followed by the engagement of regenerative processes necessary for tissue repair, which include de-differentiation and proliferation programmes that have been implicated in cancer progression.^34,35^ As a result, comparisons between cancer progression and wound healing have long been drawn, although these have largely focused on cellular responses to the physical disruption imposed by tumours on their local microenvironment.^36–38^ Over the past few years, however, genomic instability as a result of ongoing errors during mitosis (described below) has emerged as a chronic and tumour cell-intrinsic source of inflammatory signalling that can drive metastasis.^31^ Given the inherently destructive nature of inflammation, it is surprising that genomically unstable tumour cells have not only adapted to this chronic inflammatory stimulus by suppressing its immune-mediated consequences,^39^ but also actively engage its response pathways to drive metastatic progression.^32^ In this section, we explore how the stark contrast in outcome between wound healing and cancer metastasis results in part from the nature of the inflammation itself: wound healing is characterised by a short-term acute response, whereas the inflammatory signals that emanate from metastatic cells are driven by a chronic, unresolved stimulus. We also discuss how responses to acute and chronic stimuli have been linked to differences in stress response pathways and the immune-regulatory activity of nuclear factor-κB (NF-κB).

### Wound healing is initiated by a transient acute inflammatory response

As mentioned above, the lung has remarkable regenerative capabilities despite being a relatively quiescent organ.^24^ Its constitutive stem and progenitor lineages must rapidly respond to commonplace injuries caused by exposure to pathogens, toxins, pollutants, irritants and allergens.^40^ As with damage to any tissue, the wound healing process begins with inflammation to clear cellular debris and resist infection. In response to acute lung injury, cellular damage signals are rapidly induced to demarcate epithelial barrier breach. These damage signals include calcium waves, reactive oxygen species (ROS), purinergic signalling, cytokines and chemokine signalling,^21,41^ which stimulate the migration of neutrophils from the alveolar capillaries as an immediate defence against bacterial dissemination.^42^ Neutrophil migration induces elastase-mediated cleavage of E-cadherin, which directly activates β-catenin signalling to initiate regeneration of the lung epithelium.^43^ Additionally, neutrophils clear cellular debris from damaged tissues and prevent pathogen infection. Neutrophils have been shown to produce web-like DNA structures called neutrophil extracellular traps (NETs).^44,45^ These entrap foreign bodies in the wound, such as extracellular bacteria, and further stimulate and recruit immune cells that promote the clearance of dying cells.^46^ In the context of cancer, infiltration of neutrophils in the tumour microenvironment (TME) has been linked to the acquisition of metastatic traits.^47^ It is also entirely possible that NETs can promote tumour growth^48^ and metastasis.^49^ NETs can catch circulating tumour cells, promoting the formation of micro-metastasis in areas with NET deposition. Additionally, NETs have been demonstrated to awaken dormant tumour cells through integrin activation and FAK–ERK–MLCK–YAP signalling.^50^

Clearance of debris from damaged cells in the lung is aided by alveolar macrophages and the recruitment of mononuclear-lineage phagocytes such as monocytes. Macrophages have been described along a continuum of pro-inflammatory and anti-inflammatory states.^51^ Early macrophage populations are typically pro-inflammatory (often referred to as M1) and express the interleukins IL-6 and IL-1β, as well as the cytokines tumour necrosis factor-α (TNF-α) and CC chemokine ligand 2 (CCL2). These M1 macrophages sustain the initial inflammatory response by releasing toxic species, such as ROS, and matrix metalloproteinases (MMPs) which help to break down the ECM.^52^ They also stimulate further neutrophil recruitment through the release of chemokines. This positive feedback ends when the macrophage population progresses to a predominantly anti-inflammatory state (often referred to as M2). This phenotypic shift in the tissue microenvironment is triggered by macrophage engulfment of apoptotic cells and is enhanced by the increased expression of ‘eat me’ signals such as the mannose receptor, which enhances efferocytosis (the immunologically quiescent mechanism of apoptotic cell removal) and provides positive feedback to drive further engulfment of apoptotic cells.^53^ These M2 macrophages provide negative feedback on the inflammation response, both by reducing the expression of pro-inflammatory cytokines that recruit additional neutrophils and by removing existing apoptotic neutrophils through efferocytosis. This marks a transition from an inflammatory to a reparative phase, an important step in lung regeneration that is necessary for repair. Disruption of these feedback controls leads to the failure to transition from the inflammatory to the reparative phase and subsequent inappropriate remodelling of the ECM, resulting in the formation of a fibrotic scar and/or emphysematous pathology.^54^

### Chronic inflammation triggered by genomic instability in metastasis

Genomic instability^31^ is a tumour cell-intrinsic source of chronic inflammation that has been linked to aggressive features of cancer such as therapeutic resistance and metastasis.^31,55^ By virtue of its ability to induce inflammatory signalling through sensing of double-stranded DNA (dsDNA) in the cytosol, genomic instability can provoke the aberrant engagement of wound healing processes related to migration, regeneration and proliferation. However, this inflammation is not subject to the feedback mechanisms that regulate acute inflammatory responses because the source of inflammation—genomic instability within the tumour cell itself—is never resolved.

**Fig. 3:**
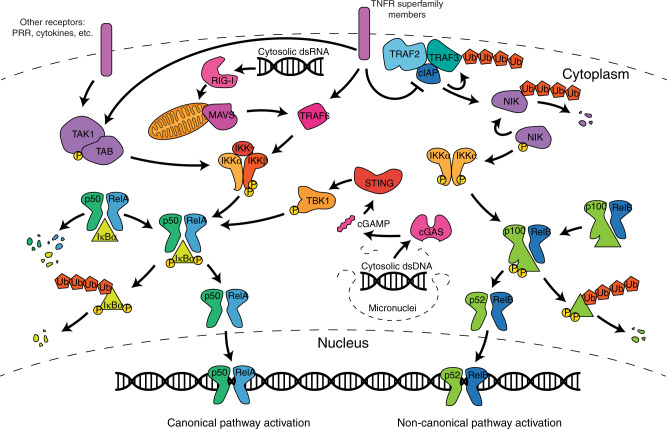
NF-κB signalling cascade. NF-κB is made up of both a canonical and non-canonical pathway, consisting of p50/RelA and p52/RelB, respectively. In the canonical pathway, TNFR superfamily members, as well as other transmembrane receptors, activate TAK1/TAB. TAK1/TAB then phosphorylates IKKβ, which in turn phosphorylates IκBα. IκBα acts to block the nuclear localisation domains of p50/RelA. However, when IκBα is phosphorylated by IKKβ, it dissociates from p50/RelA and is ubiquitinated and degraded. This frees p50/RelA to enter the nucleus where they can activate NF-κB1 responsive genes. In the non-canonical pathway, TNFR superfamily members deactivate the cIAP–TRAF2–TRAF3 complex. NIK is constantly being produced in the cell and is typically maintained in a steady state by degradation by cIAP. When cIAP is inactivated, NIK is allowed to accumulate in the cell. This causes it to phosphorylate IKKα, which in turn phosphorylates p100. This causes p100 to be processed into p52, which exposes the p52/RelB nuclear localisation domains. They can then enter the nucleus to activate NF-κB2 responsive genes. These pathways can also be activated by sensing other stimuli in the cell, such as dsRNA or dsDNA. These are sensed by the RIG-1 pathway or the cGAS-STING pathway, respectively.

The presence of dsDNA in the cytosol is a strong damage signal that indicates infection or corruption of the mitochondria or nucleus.^56^ dsDNA is sensed by cyclic GMP-AMP synthase (cGAS), which catalyses the formation of cGAMP, a cyclic dinucleotide. Under normal conditions, cGAMP promotes the activation of stimulator of interferon genes (STING)–IRF3-induced type I interferon (IFN) signalling, which provokes antigen presentation (during infection and cancer) through dendritic cell intermediaries to prime CD8^+^ T cells.^57^ The type I IFN response can be a potent chemotactic agent for many immune cells, such as natural killer (NK) and T cells; and it has been shown to exhibit strong anti-tumour effects.^58^ Furthermore, the secretion of cGAMP by mammalian cells (including cancer cells) into the extracellular space, or its transfer through gap junctions, allows for direct engagement of STING in surrounding antigen-presenting cells. Paracrine interactions driven by this can indeed fuel IFN induction in the TME, which has been shown to mediate immune cell killing of cancer cells.^59^ However, recent work demonstrates metastatic cancer cells have evolved to degrade extracellular cGAMP through expression of the ectonucleotidase ENPP1 to suppress the tumour-extrinsic effects of this immune-stimulatory metabolite.^39^ STING activation also triggers the IKK-mediated phosphorylation of IκB, which allows for NF-κB translocation to the nucleus.^57^ NF-κB exists in two forms (Fig. 3), p50 and p52, each of which can form homodimers which function as transcriptional repressors, or can heterodimerise with RelA and RelB respectively to activate transcription. The identity of the heterodimers dictates whether the canonical (p50/RelA) or non-canonical (p52/RelB) pathway is activated. The molecular pathways that link NF-κB and inflammation have been reviewed in depth.^60^

Genomically unstable cancer cells, arising from ongoing chromosome segregation errors during mitosis, generate rupture-prone micronuclei that expose genomic dsDNA to the cytosol. How these cells tolerate constitutive cGAS-STING activation without eliciting an anti-tumour immune response appears to hinge on the IFN *dependent* and *independen*t activities of STING, which are engaged by the canonical and non-canonical pathways of NF-κB respectively. Bakhoum et al. demonstrated that metastatic cells with genomic instability lack an IFN response and activate NF-κB through the non-canonical pathway.^31^ Wu et al. showed that a single point mutation in STING (S365A) specifically abrogates IFN signalling to promote immune evasion.^61^ Immune evasion requires balancing surveillance from both NK cells and CD8^+^ cytotoxic T cells. MHC class I expression is reduced in many cancer cells^62^ to avoid the risk of presenting tumour antigens to CD8^+^ T cells. However, its absence instead contributes to the activation of NK cell-mediated cytotoxic effects when activating receptors on NK cells are concomitantly engaged. For example, NKG2D on the surface of NK cells can interact with MICA/B glycoproteins present on the surface of tumour cells to trigger NK-mediated cytolysis.^63^ Although NKG2D ligands are typically upregulated in non-tumour cells by genotoxic stress and stalled DNA replication, it remains unclear whether NK activating ligands are regulated by genomic instability.^64^ Interactions between other NK activating and inhibitory ligands have been extensively reviewed.^65^ NK cells have been shown to be particularly responsive to paracrine cGAMP signalling, and might further recruit CD103^+^ stimulatory dendritic cells to repress tumour progression and metastasis through enhanced antigen presentation to T cells.^66,67^ This represents a critical trade-off that tumour cells must overcome: do they express MHC-I and risk clearance from cytotoxic T cells, or do they repress MHC-I and become more susceptible to NK cell-mediated killing?

Genomically unstable cancer cells can mitigate the deleterious, immune-mediated consequences of tumour cell-triggered inflammation, in part through suppression of the type I IFN response.^31^ Indeed, tolerance of continuous genomic segregation errors likely represents an evolutionary bottleneck that ultimately promotes both cellular invasion and metastasis.^31^ Unlike normal cells, cancer cells largely respond to chronic cGAS-STING activity through the non-canonical p52/RelB NF-κB pathway,^57^ which critically lacks type I IFN signalling capacity. This may indicate why inactivating mutations in the cGAS-STING pathway are rarely observed in cancer as a mode of immune evasion despite its role in primary tumour surveillance.^32^ Alternatively, tumour cells might co-opt the STING-dependent DNA-sensing pathway in a manner that maintains NF-κB signalling but does not induce type I IFN, through an increase in the expression of immune checkpoint inhibitors such as PD-L1,^68^ recruitment of immune-suppressive regulatory T (T_REG_) cells^69^ or loss of downstream IRF3.^70^ Maintaining NF-κB signalling is relevant in cancer cells, as its expression has been shown to promote metastasis through induction of TWIST and the epithelial–mesenchymal transition (EMT).^71^ In addition to tumour cell-intrinsic effects, the cGAS-STING pathway can also engage in crosstalk with non-immune neighbouring cells, such as astrocytes, in metastatic breast cancer.^57^ The transmission of cGAMP, generated by tumour cGAS to adjacent astrocytes through gap junctions, activates STING in astrocytes to stimulate the release of inflammatory cytokines such as TNF-α. The release of TNF-α and IL-6 can subsequently promote metastasis through the enrichment of NF-κB-driven EMT and stem cell programmes. In this way, metastatic cells can exploit the pro-metastatic effects of cGAS-STING signalling without triggering an anti-tumour type I IFN response.

dsDNA is not the only nucleic acid that can trigger cellular inflammation when detected in the cytosol. Cytosolic dsRNA is also recognised by mammalian cells as a potential virus through the dsRNA-binding proteins RIG-I and MDA5.^72^ Interestingly, paracrine activation of RIG-I is associated with resistance to anti-cancer therapies and increased metastasis.^73^ RIG-I activation can regulate the levels of canonical NF-κB at a post-transcriptional level by binding to the *NFKB1* mRNA at three distinct tandem motifs recognised within its 3′-UTR.^74^ RIG-I has also been shown to be activated by RN7SL1, an abundant RNA that acts as a scaffold for this pattern recognition receptor.^75^ Typically, RN7SL1 is shielded by the RNA-binding proteins SRP9 and SRP14, which prevents its 5′ end from being detected by RIG-I. However, excess, unshielded RN7SL1 can pass from stromal fibroblasts to breast cancer cells through exosome-mediated crosstalk, thereby promoting an inflammatory microenvironment and enhancing metastasis. A similar response can be induced by retroelements, such as endogenous retroviruses and retrotransposons,^76^ whereby tumour exosomes containing transposable elements are taken up by stomal fibroblasts to elicit a pro-metastatic inflammatory response.^77^

Although the source of aberrantly localised nucleic acids in both systems is vastly different, the outcomes are the same—cytosolic nucleic acid sensing triggers an inappropriate and chronic inflammatory response. Understanding how sensing of cytosolic nucleic acids promotes metastasis in some contexts and anti-tumour effects in others is essential to its effective therapeutic targeting.^78^ It is plausible that the divergent functional responses reflect chronic versus acute pathway activation.

### Engagement of the non-canonical NF-κB pathway enhances tumour cell migration

Tumour cell-intrinsic inflammatory signalling can lead to persistent NF-κB activation through canonical and non-canonical pathways. As outlined above, chromosomally unstable cancer cells show increased activation of the non-canonical pathway,^31^ which is slow and persistent—in stark contrast to the rapid and transient canonical response.^79^ Identifying regulators of non-canonical NF-κB signalling and how they influence chromosomally unstable cellular phenotypes could provide avenues for therapeutic intervention. Although the link between metastasis, NF-κB and EMT was initially established through canonical RelA:p50 NF-κB signalling, the non-canonical pathway is notably active in migratory CD103^+^ dendritic cells^80^ and is required for the migration of macrophages in response to HMGB1, an alarmin that activates inflammatory pathways within immune cells.^81^ The non-canonical RelB/p52 complex can also induce cyclin D1 and c-MYC promoters, which control the cell cycle and promote proliferation.^82^ It is therefore possible that tumour cells co-opt the promigratory function of non-canonical NF-κB signalling, typically seen in dendritic cells of the immune system, for metastasis. However, the molecular pathways underlying this functional mimicry in cancer metastasis remain unclear.

## Regeneration

Upon initiating immune cell responses to injury to prevent infection, inflammatory signalling must trigger compensatory regeneration of lost and/or damaged cells to restore tissue function. However, such processes must be tightly controlled to prevent either too little or too much growth. The former would lead to an open, persistent wound that is susceptible to infection, whereas the latter leads to scarring, fibrosis and cancer.^24,83^ Likewise, inflammatory signals emanating from tumour cells themselves or their microenvironment can engage tumour stem and progenitor cell functions to (re)generate metastases at secondary sites (Table 1).^84^ These cancer cells take on stem-like characteristics as they undergo EMT and engage with developmental programmes, which are often regulated by the Sox family of transcription factors. In this section, we explore the dynamic interplay between regeneration and immunity in both wound healing and metastasis, with a focus on activators of stemness, such as β-catenin and transforming growth factor-β (TGF-β). The engagement of developmental pathways in stem-like cancer cells increases their migratory and invasive features, whilst also shaping their immune susceptibilities.^13^ Understanding how this interplay contributes to the plasticity of DTCs—especially phenotypic switching between proliferation, migration and differentiation states—is critical for developing treatments that could effectively target lethal, metastasis-initiating stem cells.

**Table 1:** Key cell types and mediators in wound healing and metastasis.

Cell type	Key mediators	Role in wound healing cascade	Role in metastasis
B cells	IL-6, IL-10	Secrete antibodies. Can acclerate wound healing by increasing fibroblast proliferation and decreasing apoptosis in wound bed	A subset of IL-10-producing regulatory B cells are able to promote metastasis by suppressing cytotoxic CD8^+^ T cells
Dendritic cells	IL-10, IL-12, E-cadherin, MIP-1α, CCL2	Present antigens to T cells to activate the adaptive immune response. Subtypes are able to express CD103. In the presence of integrin B7, CD103^+^ dendritic cells can bind e-cadherin through the AlphaE–Beta7 complex important for tissue retention factor	Repress tumour progression through antigen presentation to T cells and activation of cancer immune surveillance
Macrophages (M1) pro-inflammatory	TGF-β, TNF-α, IFN-γ, IL-1β, IL-1RA, IL-6, IL-10, IL-12, CXCL1/2/3, MIP-1α, CCL2	Phagocytose dead cells and pathogens. Express TNF-α and interleukins IL-6 and IL-1β to further stimulate immune response. Remove excess fibroblasts and extracellular matrix	Repress tumour progression by inducing apoptosis and senesence
Macrophages (M2) anti-inflammatory	TGF-β, TNF-α, IL-1β, IL-1ra, IL-6, IL-10, IL-12, EGF, KGF, FGF, VEGF, CXCL1/2/3, MIP-1α, CCL2	Promote new blood vessel formation through the release of growth factors such as vascular endothelial growth factor (VEGF). Actively signal dermal fibroblasts to regenerate the ECM by increasing collagen and actin deposition	Produce growth factors and inflammatory cytokines that promote stemness in cancer cells
Mast cells	IL-1β, IL-10	Release pro-inflammatory cytokines, link innate and adaptive immune responses	Can release proteases into microenvironment which degrade extracellular matrix and allow metastatic invasion. The release of proteases and growth factors also promotes angiogenesis to support new tumour growth
Natural killer cells	TNF-α, IFN-γ, IL-10, IL-12	Produce IFN-γ to mediate wound healing, which activates macrophages and induces expression of MHC	Repress tumour progression through targeted killing of cancer cells
Neutrophils	TGF-β, TNF-α, IL-6, IL-8, IL-10, IL-12, CXCL1/2/3, MIP-1α	Destroy infectious threats. Deposit extracellular traps to capture foreign bodies. Enter injured tissue and break down extracellular matrix, release proteases such as cathepsin G and protease 3	Release proteases and NETs which break down the extracellar matrix of the tumour microenvironment. Promotes further inflammation and can awaken dormant micro-metastasis
T cells	TNF-α, IFN-γ, IL-6, IL-10, IL-12, EGF, KGF, FGF, VEGF, E-cadherin, CCL2	γδ+ T cells survey for ligands such as SKINTs and CD100 during epidermal stress. Release growth factors such as KGF-1, KGF-2, and insulin growth factor-1. αβ+ T cells consist of CD4^+^ helper cells, CD8^+^ killer cells and T_REG_ cells important in pathogen defense and the immune response. Immune-suppressive T_REG_ and pro-inflammatory Th17 cells balance adaptive immune response	CD8^+^ cytotoxic T cells repress tumour progression by inducing apoptosis in cancer cells. T_REG_ and Th17 cells promote and maintain an immunosuppressive and pro-tumour inflammation environment that drives metastatic progression
Alveolar epithelial cells type I (AEC1)	EGF, KGF, FGF, VEGF, CXCL1/2/3	Form the gas exchange surface in the alveolus	The epithelial–mesenchymal transition causes epithelial cells to take on stem cell like characteristics critical to metastasis such as increased migration and evasion/tolerance of immune cells
Alveolar epithelial cells type II (AEC2)	EGF, KGF, FGF, VEGF, CXCL1/2/3	Secrete pulmonary surfactant to reduce surface tension. Able to proliferate and differentiate into AEC1	
Alveolar epithelial progenitor (AEP)	TGF-β, EGF, KGF, FGF, VEGF, CXCL1/2/3	Regeneration of a large proportion of the alveolar epithelium, including AEC1 and AEC2	
Endothelial cells	EGF, KGF, FGF, VEGF	Line the surface of blood vessels. Respond to hypoxic responsive growth factors such as VEGF and PDGF. Break down the ECM, proliferate and migrate to form new capillaries	Form a barrier to prevent metastatic cancer invasion. Can promote macro-metastasis through angiogenesis
Fibroblasts	TGF-β, IL-8, CXCL1/2/3	Deposit and remodel ECM and granular tissue. Act as a scaffold for immune cells and new blood vessels. Help to contract the wound	Produce growth factors, proteolytic enzymes and ECM components that may determine pre-metastatic niche formation
Myofibroblasts	TGF-β	Express α-SMA, as well as β- and γ-cytoplasmic actins. α-SMA is recruited to stress fibres at the fibronexus freeing TGF-β1 from its latent complex	
Pericytes		Regulate blood flow, form a vascular barrier to bacteria and stabilise the microvasculature	Restrict primary tumour cell escape Contribute to pre-metastatic niche

The wound healing response involves interplay between a diverse number of epithelial and immune celltypes (recently reviewed in-depth^21^); many of these cell types are also implicated in the progression of cancer metastasis. These cell types interact through various pro-inflammatory and anti-inflammatory cytokines, chemokines and growth factors.

### Epithelial regeneration in wound healing is mediated by bidirectional crosstalk with the immune microenvironment

The regenerative phase of wound healing is marked by a transition of the macrophage population from the M1 to the M2 state. Consequently, the release of pro-inflammatory cytokines and chemokines such as CCL2 is replaced by the production of growth factors and anti-inflammatory cytokines including TGF-β, IL-10, vascular endothelial growth factor (VEGF), and hepatocyte growth factor (HGF), which promote the proliferation of lung parenchymal cells.^52^ These growth factors are produced by both M2 macrophages and helper T cells, like T_H_17 cells, which also release large amounts of IL-22, a host defence cytokine that also promotes epithelial proliferation (reviewed in depth elsewhere.^85^) The influx of growth factors leads to the activation of proliferation and differentiation programmes necessary for epithelial repair and metastatic progression. They have also been shown to mediate crosstalk between epithelial cells and macrophage^86,87^ or T_H_17^88,89^ populations. In the lung, this repair process involves several facultative progenitors: which are specialised cell types that can be induced upon injury to proliferate and differentiate into one or more cell types as part of a regenerative response.^90^ For example, in the alveolar compartment, the release of TGF-β from M2 macrophages triggers the replacement of damaged type I alveolar epithelial cells (AEC1) with proliferating type II alveolar epithelial cells (AEC2).^91^ Typically, AEC1 form the gas exchange surface in the alveolus^92^ and AEC2 secrete pulmonary surfactants that reduce surface tension to prevent alveoli collapse.^54^ While AEC2s were long thought to be the exclusive source of postnatal AEC1s, cell lineage-tracing studies have since demonstrated that multiple progenitor and stem cell populations can play a context-dependent role in lung regeneration.^93^ These populations include a conserved alveolar epithelial progenitor (AEP) marked by the expression of transmembrane 4 L6 family member 1 (TM4SF1), which has been shown to repopulate a large portion of the alveolar epithelium^94,95^ through signalling involving WNT and fibroblast growth factor (FGF)^96^ and is enriched in metastatic cells.^13^ While the nature of the spatiotemporal signals that trigger specific progenitor cell responses remains unclear, single-cell approaches are poised to reveal how distinct stem and progenitor lineages integrate in both wound healing^97^ and cancer progression.^13^

In parallel with epithelial regeneration, fibroblasts are stimulated by macrophages to migrate to the injury site, where they proliferate and synthesise new ECM and granular tissues. Some fibroblasts will acquire a more contractile phenotype by expressing α-smooth muscle actin (SMA), as well as β- and γ-cytoplasmic actins.^21,98^ Within these myofibroblasts, α-SMA is recruited to stress fibres at sites called the fibronexus.^21^ The force exerted by these stress fibres frees TGF-β1 from its latent complex and into its active form, which can promote the generation of more myofibroblasts in a positive feedback loop.^99^ Once tissue integrity is restored, myofibroblasts are ultimately cleared from wound sites via apoptosis. The failure to clear myofibroblasts can lead to scarring conditions, including many forms of fibrosis.^21^

### The positive interplay between inflammation and stemness is mediated by NF-κB activation

Tumour de-differentiation is partially mediated through NF-κB crosstalk with neighbouring macrophages. Macrophages are abundant at the invasive front of many advanced cancers^86,100^ and M2 macrophages produce many growth factors and inflammatory cytokines that promote stemness in lung cancer cells, including EGF, TGF-β, IL-1, IL-6, IL-10 and TNF-α.^100^ Furthermore, the depletion of M2 macrophages reduces seeding of lung metastasis.^101,102^ In contrast, M1 macrophages can decrease lung cancer viability by inducing apoptosis and senescence.^103^ The p50 subunit of NF-κB is a key regulator of the M2-driven macrophage inflammatory response in vitro and in vivo.^104^ NF- κB expression is also a key feature of stem-like cancer cells, which constitutively exhibit higher NF-κB activation^105^ in addition to developmental programmes like WNT signalling. NF-κB has been shown to maintain an undifferentiated state in lung cancer cells; its inhibition reduces cancer stem cell function.^106^ Cancer cell stemness is potentially maintained through the upregulation of LIN28B and TCF7L2 by the inflammatory factor IKKβ, a kinase member of the NF-κB pathway.^107^ Altered Toll-like receptor (TLR) signalling may also contribute to NF-κB activity and the responsiveness of stem-like cancer cells to inflammation.^105^ For example, TLR3 activation promotes tumour cell expression of the core stem cell regulators OCT3/4, NANOG and SOX2.^108^ The activity of these core stem cell regulators is maintained by NF- κB signalling.^106^ Reciprocally, SOX2-expressing cancer cells have been shown to recruit tumour-associated M2 macrophages with increased NF-κB and Stat3 activation.^109^ These tumour-associated macrophages have been shown to facilitate invasion and metastasis, and are correlated with a poor prognosis of solid tumours.^86,110^

We used single-cell RNA sequencing to demonstrate that tumour cells within lung cancer metastases exhibit a continuum of stem–epithelial progenitor states, driven largely by key endoderm- and lung-specifying SOX transcription factors.^13^ The activation of inflammatory response pathways was highly variable across these tumour developmental states, with *SOX2* enriching for migratory stem-like cells that retain inflammatory response programmes and the long-term potential to initiate metastatic outgrowth.^111^ Indeed, the expression of *SOX2* has been associated with increased motility, metastasis and chemotherapy resistance in cancer,^112,113^ and the stable knockdown of *SOX2* reduces micro-metastatic seeding in our model of delayed lung cancer metastasis.^8^ Interestingly, this suggests that inflammatory response signalling is dependent, in part, on the differentiation status of tumour cells themselves. Thus, activation of TLR–NF-κB signalling by tumour cell -extrinsic and -intrinsic factors could amplify the positive interplay between inflammation and stemness in cancer progression, potentially expanding the pool of metastasis-initiating stem cells.

### Developmental signalling pathways promote the proliferation of metastasis-initiating stem cells

Signalling pathways that are used during development form the crux of cellular decisions to migrate, proliferate or differentiate during lung regeneration and repair. Likewise, metastatic cells can engage with similar pathways to switch between these functions at new sites. Cytokines such as TGF-β, largely produced by immune and stromal cell types during wound healing in the TME, play a critical role in the regulation of tissue homoeostasis and cancer progression.^114^ A detailed summary of the TGF-β signalling pathway and its role in metastasis is available elsewhere.^115,116^ Briefly, this pleiotropic growth factor inhibits the proliferation of normal epithelial cells and cancer cells in early-stage disease, but directly stimulates the proliferation of malignant and stromal cells later in tumour progression. Additionally, TGF-β may have immunosuppressive effects that lead to T cell exclusion from tumours.^117^ In line with these observations, inhibition of TGF-β enhances the cytotoxic T cell response against tumour metastasis.^118^ TGF-β can also activate a number of signalling pathways, including Ras–mitogen-activated protein kinase (MAPK) and phosphatidylinositol 3-kinase (PI3K)–AKT, that modulate the EMT programme, independent of its ability to signal to SMAD proteins.^119^ The mechanisms underlying the shift of TGF-β from tumour suppressive to promoting roles are not well understood, but is thought to involve mutations in the mediators of TGF-β and changes to the TME that lead to dose-dependent effects of TGF-β.^120^

Metastasis-initiating cells enriched for the core stem cell transcription factor *SOX2* can self-impose a quiescent and immune-evasive state through autocrine expression of DKK1, an inhibitor of the WNT signalling pathway.^8^ Notably, these cells show increased TGF-β signalling when in a quiescent state, accompanied by reductions in WNT, MYC and NF-κB signalling.^8^ WNT is a potent mitogen for stem and progenitor cell pools that has been implicated in both tumour initiation and the metastatic progression of lung^121^ and breast^122,123^ cancers. This implies a potential trade-off where metastatic cells must be either highly invasive or highly proliferative, but not both. These contrasting cell states are perceived differently by the immune system, with highly proliferative cells more subject to immune surveillance.

### Stem-like cancer cells show increased migratory and invasive features

Disruption of epithelial integrity is a defining feature of metastasis-initiating stem cells,^124^ which must detach from their local microenvironment to disseminate systemically. The E-cadherin–β-catenin–α-catenin complex typically interacts with the actin cytoskeleton to maintain cell polarity and cell adhesion. This interaction can be disrupted through the dissolution of adherens junctions, loss of apical–basal polarity, downregulation of epithelial surface markers, reorganisation of the actin cytoskeleton, or induction of a mesenchymal gene-expression programme.^125^ Such features are typically described as part of a TGF-β-driven EMT programme, which facilitates the translocation of circulating tumour cells to distant sites during metastasis and has been extensively reviewed elsewhere.^126^ TGF-β can induce WNT signalling to promote EMT and tumour metastasis through a number of mechanisms previously reviewed in depth.^127^ During embryonic development, WNT signalling controls body axis patterning, cell-fate specification, cell proliferation and cell migration, and WNT ligands are expressed in the early stages of wound healing to promote the migration of epithelial cells.^34^ During lung metastasis, differentiated cells can acquire stem-like properties through combined activation of WNT and NF-κB signalling, as described above.^128^ This can occur in a spatially restricted manner, whereby tumour cells located at the invasive front of a tumour express WNT target genes that confer an invasive-metastatic capacity that is not shown by well-differentiated cells located in the centre of the tumour.^128^ Cells at the invasion front with improperly activated WNT signalling undergo EMT, which involves the displacement of β-catenin from adherens junctions. Its translocation to the nucleus then drives the expression of molecules such as CD44, which are associated with cancer stem cell traits. This implies that cancer cells at the leading edge of an invasive tumour that contain activated β-catenin could indirectly resist immune attack.^37^ The activation of Wnt/β-catenin in tumour cells can also function in immune evasion by inhibiting the recruitment of CD103^+^ dendritic cells, which are critical for adaptive immune recognition of the tumour.^14,129^

### Tumour cell differentiation status is coupled to innate immune surveillance

NK cell-mediated killing plays a critical role in restricting the outgrowth of DTCs^130^ in a lineage- and cell cycle-dependent manner.^13,131^ Tumour cells with reduced lineage differentiation and greater stem-like attributes show distinct susceptibilities to NK cell-mediated killing in a cell cycle-dependent manner.^36,132–134^ For example, although DTCs distinguished by *SOX2* expression were required to seed metastases,^8^ they were also more sensitive to NK cell-mediated clearance.^13^ Immune escape was only facilitated in a quiescent subset of these metastasis-initiating stem cells through increased MHC class I expression.^13^ The induction of quiescence, through expression of the WNT inhibitory protein DKK1, led to broad downregulation of NK cell-activating ligands and evasion of immune surveillance.^8^ Conversely, more differentiated lung epithelial progenitors (specified by *SOX9* expression) displayed increased resistance to NK cell-mediated killing in a spontaneous model of metastatic escape in vivo and upon co-culture with IL-2-activated NK cells in vitro, regardless of proliferation status. Depletion of NK cells facilitated the emergence of diverse cancer cell subpopulations during metastasis, including the outgrowth of phenotypic states that were otherwise suppressed.^13^ Likewise, polyclonal metastases arising from circulating tumour cell clusters with increased epithelial characteristics also exhibited a higher resistance to NK cell-mediated killing.^124^ Perturbation of epithelial status directly altered the expression of NK ligands by tumour cells and increased susceptibility to NK cell killing.^124^ Therefore, tumour cell differentiation status and epithelial and mesenchymal features, as well as acquisition of a slow-cycling state, play crucial roles in NK cell-mediated control of metastasis. This may be especially important in restraining the outgrowth of solitary, mesenchymal stem cells that can lie dormant for months to years as latent entities before giving rise to overt metastases.

### A phenotypic trade-off between tumour cell migration and proliferation

Aggressive tumour cells display a phenotypic trade-off between migration and proliferation during the invasion-metastasis cascade. This apparent trade-off was first observed in highly aggressive brain cancers,^135^ and has led to a number of papers and mathematical models that either support or reject a ‘Go-versus-Grow’ hypothesis. A transcriptomic characterisation of 500 metastatic solid tumours of diverse lineage and tissue origin likewise exhibited two dichotomous meta-states that could be characterised by either increased proliferation and metabolism or increased mesenchymal traits and inflammatory response programmes.^136^ Compromises only become apparent when both phenotypes can no longer be optimised, perhaps explaining why this trade-off is not apparent in all tumour types.^137^ The optimal state between multiple traits is often described as Pareto optimality,^138^ a concept that is often used in engineering and economics to describe maximally efficient resource allocation. This concept can also be applied to biological systems like cancer. Tumour cells that have not reached Pareto optimality do not necessarily display an inverse correlation between linked traits; instead, traits can appear to be unrelated^139,140^ or even positively correlated.^140^ This concept may help to explain why the dichotomous relationship between migration and proliferation was first observed in particularly aggressive tumours. The switch between these two phenotypes does not seem to be mutationally driven, but instead is driven by changes to the TME.^141^ For example, ECM proteins that facilitate cell migration, such as merosin, have been found to increase the lag phase of growth.^135^ Similarly, a hypoxic environment favours the emergence of the invasion phenotype.^141^ Phenotypic switching has important implications for treatment, as most radiation and chemotherapies target highly proliferative cells and, as such, are much less effective against DTCs with mesenchymal stem-like traits, which are primed to enter a slow-cycling state and ultimately seed lethal metastases.

## Remodelling

Remodelling constitutes the final stage of wound healing; however, it is important to note that this stage is not fully achieved during cancer progression because genomic instability remains an unresolved source of tumor inflammatory signaling. Nevertheless, tumour cells can actively sequester the immune-stimulatory effects of cGAS-STING pathway activity away from the TME^39^ and processes involved in tissue remodelling can facilitate DTC invasion, dissemination and colonisation. In this section, we discuss how wound remodelling involves a switch from a regenerative microenvironment back to an inflammatory one in order to clear excess collagen and other materials from the now-healed wound. If the wound healing process is interrupted or delayed, remodelling can fail and lead to fibrosis, taking on many characteristics observed in metastases.

### Wound remodelling clears excess cells and ECM produced during regeneration

When epithelial integrity has been properly restored, the final phase of wound healing is characterised by downregulation of the reparative pathways initiated after injury.^142^ Most cells that were recruited to the wound site undergo apoptosis or migrate away from the injury, leaving behind mainly collagen and other ECM components. Macrophages regain their phagocytic characteristics to remove fibroblasts and matrix that are no longer required.^21^ This process is aided by the secretion of proteases, such as cathepsins, MMPs and plasmin by resident macrophages.^143^ Macrophages may also play a role in restoring epithelial tight junctions during alveolar repair. IL-1 receptor antagonist (IL-1RA) expressed by macrophages has been reported to act as an antagonist to IL-1β, thereby stabilising the tight junction protein ZO-1.^144^ Upon restoration of alveolar barrier function, oedema is cleared by active sodium transport through epithelial Na^+^ channels. This process is initially repressed by pro-inflammatory mediators such as IL-1β and TNF-α, but can be subsequently restored by the growth factors keratinocyte growth factor (KGF) and EGF produced by T_REG_ cells.^145^ Such growth factors further stimulate the expansion of progenitor cells to restore lung function.^146^

### Failed remodelling caused by delayed inflammation leads to fibrosis

Pulmonary fibrosis can result from incomplete wound healing; for example, in the context of persistent exposure to harmful substances, viral infections, or in scleroderma: a chronic connective tissue disease.^143^ Dysfunction in the wound healing response leads to the accumulation of ECM, which prevents proper lung function and can ultimately lead to respiratory failure. Fibrosis is generally characterised by injury to alveolar epithelial cells, activation of fibroblasts and myofibroblasts, overproduction of ECM, and dysregulation of macrophages.^22^ Delaying the production of chemokines and cytokines in wounds is a common source of chronic inflammation that can lead to non-resolving wounds.^21^ For example, Monocyte chemoattractant protein-1 (MCP-1)/CC chemokine ligand 2 and macrophage inflammatory protein 2 (MIP-2)/CXCL2 (chemokine (C-X-C motif) ligand 2) are necessary for the influx of monocytes and activation of macrophages. When macrophage activation is delayed, the removal of apoptotic cells by efferocytosis is impaired and accumulated apoptotic cells undergo secondary necrosis. Residual apoptotic and necrotic cellular debris leads to the persistence of neutrophils in the wound and activation of damage-associated molecular pattern (DAMP) receptors, which trigger a chronic inflammatory response (reviewed in depth in ref. ^147^) This can lead to the unchecked release of proteases by activated neutrophils, which further break down the wound microenvironment. Inflammation can persist into the remodelling stage, thereby preventing wound healing. Prolonged lung inflammation often leads to an imbalance between M1 and M2 macrophages; for example, patients with idiopathic pulmonary fibrosis typically show increased proportions of M2 macrophages.^144^ M2 macrophages promote the excessive production and deposition of collagen by myofibroblasts through the secretion of factors including TGF-β, fibronectin, proline and arginase. They also release tissue inhibitors of metalloproteinases (TIMPs), which are necessary for wound resolution. The persistence of M2 macrophages into the remodelling phase prevents M1 macrophages from effectively resolving the wound by clearing excess immune cells, fibroblasts and collagen. Immature collagen and other ECM proteins in turn promote the polarisation of yet more M2 macrophages,^143^ leading to a positive feedback loop that drives the progression of fibrosis.

### Acute versus chronic injury

Failure to complete the wound healing process leads to the onset of chronic inflammation, akin to that emanating from genomically unstable tumour cells. Chronic inflammation differs from acute inflammation in both the enumeration and behaviour of cell types involved, as well as their mediators.^148^ For example, neutrophils present in wounds may damage tissues through the inappropriate release of NETs.^149^ Interestingly, NETs have been observed around metastatic tumour cells that have reached the lung; in vitro, these NETs stimulated the migration and invasion of breast cancer cells.^48^ NETs have also been reported to be involved in re-awakening dormant cancer cells, with NET-associated proteases cleaving laminin, which, in turn, activated integrin to induce the proliferation of dormant cancer cells.^150^ Chronic inflammatory signalling can also perturb the M1 and M2 macrophage balance. A prolonged M1 state driven by IFN-γ may result in the excessive release of pro-inflammatory cytokines such as TNFα, IL-1β, IL-6, IL-12, IL-15 and IL-23.^151^ Alternatively, a defective M2 macrophage response has been shown to arise in fibrosis and cancer due to a hyper-responsive subpopulation of M2 macrophages.^151^ This macrophage transcriptional response is characterised by enhanced pathway activation of IRF3 and STAT1, and is distinct from that of the canonical M1 and M2 phenotypes (reviewed in ref. ^152^) This subpopulation presents a novel therapeutic target to disrupt or prevent both fibrosis and metastasis. Disrupted macrophage dynamics leads to chronic inflammation and resumes the wound healing cycle in an amplifying feed-forward loop of inflammation and regeneration that the wound or tumour cannot resolve.

## Conclusion

The innate wound healing process is characterised by a large number of positive and negative feedback mechanisms. These regulatory mechanisms ensure that the wound healing process progresses to completion once initiated, while also preventing excess regeneration and abnormal cell growth by tightly regulating crosstalk between inflammation, stemness and migration. Inflammatory signalling generated from within tumour cells themselves results in the chronic engagement of various aspects of the wound healing process—disrupting this interplay to create an unchecked positive feedback loop that has profound consequences on the dissemination, immune surveillance and propagation of metastasis-initiating stem cells to promote cancer progression. Just as importantly, cancer progression can occur through the loss of negative feedback controls such as apoptosis pathways and immune checkpoints which are meant to stop regeneration pathways and resolve wound healing. As the initial phases of inflammation and regeneration are never fully resolved, remodelling as the final phase of wound healing is never reached.

Insights into the functional interaction between healing, immunity and metastasis have uncovered a number of molecules and processes which could be targeted to prevent metastatic tumour cell dissemination or to sensitize tumour cells to immune surveillance, including the NF-κB and TGF-β pathways. The precise molecular pathways that govern both regeneration and metastasis remain poorly understood—yet progress has highlighted the similarities between these two processes more than ever. Further benefits can only be gained by continuing to explore interactions between regeneration, immunity and cancer.

## Data Availability

Not applicable.
